# Multi-Classification of Skin Lesion Images Including Mpox Disease Using Transformer-Based Deep Learning Architectures

**DOI:** 10.3390/diagnostics15030374

**Published:** 2025-02-05

**Authors:** Seyfettin Vuran, Murat Ucan, Mehmet Akin, Mehmet Kaya

**Affiliations:** 1 Department of Information Technologies, Dicle University, Diyarbakir 21200, Turkey; 2 Department of Computer Technologies, Dicle University, Diyarbakir 21200, Turkey; 3 Electrical-Electronics Engineering, Faculty of Engineering, Dicle University, Diyarbakir 21280, Turkey; makin@dicle.edu.tr; 4 Department of Computer Engineering, Firat University, Elazig 23119, Turkey

**Keywords:** classification, skin lesion, Mpox, transformers, ViT, MAE, DINO, SwinTransformer

## Abstract

**Background/Objectives:** As reported by the World Health Organization, Mpox (monkeypox) is an important disease present in 110 countries, mostly in South Asia and Africa. The number of Mpox cases has increased rapidly, and the medical world is worried about the emergence of a new pandemic. Detection of Mpox by traditional methods (using test kits) is a costly and slow process. For this reason, there is a need for methods that have high success rates and can diagnose Mpox disease from skin images with a deep-learning-based autonomous method. **Methods:** In this work, we propose a multi-class, fast, and reliable autonomous disease diagnosis model using transformer-based deep learning architectures and skin lesion images, including for Mpox disease. Our other aim is to investigate the effects of self-supervised learning, self-distillation, and shifted window techniques on classification success when multi-class skin lesion images are trained with transformer-based deep learning architectures. The Mpox Skin Lesion Dataset, Version 2.0, which was publicly released in 2024, was used in the training, validation, and testing processes of the study. **Results:** The SwinTransformer architecture we proposed in our study achieved about 8% higher accuracy evaluation metric classification success compared to its closest competitor in the literature. ViT, MAE, DINO, and SwinTransformer architectures achieved 93.10%, 84.60%, 90.40%, and 93.71% accuracy classification success, respectively. **Conclusions:** The results obtained in the study showed that Mpox disease and other skin lesion images can be diagnosed with high success and can support doctors in decision-making. In addition, the study provides important results that can be used in other medical fields where the number of images is low in terms of transformer-based architecture and technique to use.

## 1. Introduction

Monkeypox (Mpox) is a difficult-to-detect disease whose clinical symptoms are similar to those of varicella and has become a global public health problem since 2022 [1]. Skin lesions, rashes, and high fever are important symptoms in patients infected with monkeypox virus [2]. Considering the rate of transmission of the disease and vulnerable populations, it is an important public health problem facing the world after COVID-19. The disease has spread to more than forty countries, and the travel warning level in the United States has been raised to protect against the disease. Traditional medical methods use polymerase chain reaction (PCR) and serologic testing to diagnose Mpox [3]. However, these methods are slow and resource-intensive. The recent COVID-19 pandemic has shown the impact of early diagnosis of infectious diseases on the spread of the disease [4,5,6]. Early detection of the disease can make important contributions to the protection of public health. For these reasons, it is necessary to develop autonomous systems that can detect Mpox disease in skin images.

Deep learning architectures, a subfield of artificial intelligence, have been successfully used in many areas of human life, such as medicine, engineering, and agriculture. Researchers are trying to solve many challenging tasks, such as classification [7], segmentation [8], and object detection [9], using deep learning architectures and medical images. Doctors can get support from deep-learning-based systems during the diagnosis of diseases. The support received by doctors from an autonomous system during the diagnosis of diseases can increase their diagnostic success and prevent incorrect treatments. Processing skin images with deep learning architectures and using them to diagnose Mpox disease will progress very quickly compared to traditional methods. By using them in hospitals where diagnostic tests are not available, many patients will be able to receive early diagnosis and treatment.

Studies aimed at training deep learning architectures with skin images have been recently reported by some researchers in the literature. Ali et al. focused on developing a web-based system for the detection of skin lesions and the related Mpox disease [10]. The researchers classified six classes of skin images using a self-collected and openly available dataset. They trained a total of seven different architectures using the weights of ImageNet and HAM1000 datasets. As a result of their classification using popular deep learning architectures based on convolutional neural networks, they achieved the highest success with the DenseNet121 architecture. The researchers achieved the highest classification accuracy of 81.70 using ImageNet weights and 82.26 using HAM1000 weights. Another study focusing on the detection of Mpox disease from skin images was conducted by Biswas et al. [11]. In two-class and six-class studies, the researchers tried to diagnose Mpox and other diseases, and in this sense, they conducted their research in two different branches. The two-class studies address the issue as a two-class classification problem focusing only on the relevant disease that provides information about the presence or absence of Mpox disease. They carried out the training process with a model they named BinaryDNet53 based on the DarkNet53 deep learning model. They achieved an accuracy of 95.05 for two-class classification and 85.78 for six-class classification.

Bala et al. considered monkeypox as a four-class classification problem [12]. Images labeled as chickenpox, measles, monkeypox, and normal were trained with an architecture called MonkeyNet, which was created with DenseNet blocks. The researchers achieved an accuracy of 93.19 with the four-class classification method. Another study focusing on classifying four-class Mpox images was conducted by Akram et al. [13]. The researchers achieved 90% success in the accuracy evaluation metric with their transfer learning-based architecture called SkinMarkNet.

Although two- and four-class classification studies are important in classifying images of Mpox disease, they are insufficient in identifying skin diseases. There are six different classes that can be identified from skin images. There is a need for new studies covering all skin diseases. Studies that include all of these classes do not have high enough success to be used in clinical studies. In this context, the motivation of our study is that there is still a need for a deep learning architecture that has a flexible structure and can achieve high accuracy in detecting Mpox images on six-class skin images. In addition, the studies reviewed in the literature are based on popular architectures from convolutional neural networks. However, transformer-based architectures are more suitable for use in medical images with their flexible structure. Transformer-based deep learning architectures provide advantages to researchers who want to focus on medical images due to their customizable structures, multilayer structures, and attention mechanisms.

This study aims to diagnose Mpox disease from skin lesion images using transformer-based deep learning architectures, specifically ViT, MAE, DINO, and SwinTransformer. The performances of these architectures are analyzed in detail, highlighting their strengths and weaknesses in classifying skin lesion images. The study demonstrates the superiority of transformer-based models over CNN-based architectures in diagnosing Mpox disease and provides new approaches for this application that have not been previously explored in the literature. Furthermore, the contributions of specific techniques, such as the masked autoencoder approach in MAE and the self-supervised learning method in DINO, to the classification of Mpox and other skin lesion images are extensively studied. The effectiveness of shifted windows in transducer-based architectures is also examined in the context of skin lesion classification. The study also provides insight into the relative computational costs of ViT, MAE, DINO, and SwinTransformer architectures by evaluating their computational efficiency and training time under identical conditions.

## 2. Materials and Methods

### 2.1. Dataset and Implementation Details

Mpox Skin Lesion Dataset Version 2.0 (MSLD v2.0) was used in the training and testing processes of the study [10,14]. MSLD v2.0 contains skin lesion images with six classes. The dataset contains 284, 75, 55, 66, 161, and 114 images of Mpox, Chickenpox, Measles, Cowpox, HFMD, and Healthy classes, respectively. A total of 755 original skin lesion images were obtained from 541 patients. All images in the dataset have been approved by professional dermatologists and regulatory authorities, making it a reliable dataset. The dataset is also the largest and most recent in the literature that is used for the diagnosis of Mpox images. The dataset and associated publication were published on 20 August 2024. MSLD v2.0 treats the problem as a six-class classification problem, whereas similar examples treat it as a two- or four-class classification problem.

**Figure 1:**
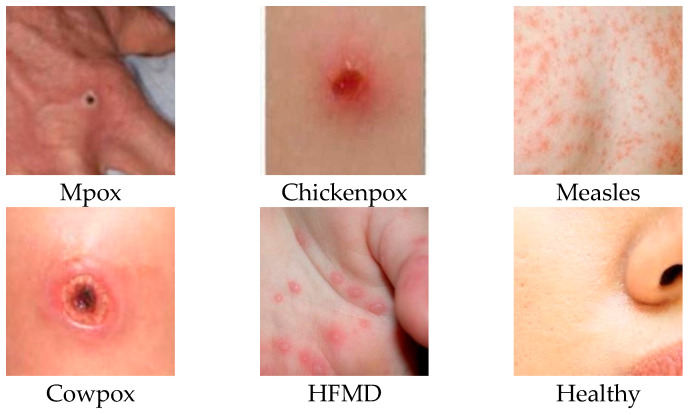
Selected images and class labels from the Mpox Skin Lesion Dataset Version 2.0.

Ali et al. [10], who collected the dataset and published it as open access, published the original versions of the images as well as the version with data enhancement techniques applied from the same source. The researchers applied various data augmentation techniques, such as rotation, translation, reflection, cut, hue, saturation, contrast, brightness flicker, noise, and scaling, to the original images. The dataset is suitable for use in transformer-based deep learning architectures due to its heterogeneous class distribution. We used the original and augmented dataset version used by the researchers who generated the dataset and published it as open access. The dataset is divided into training, validation, and testing subsets in a ratio of 70:20:10, respectively. Our motivation for using this dataset is to obtain reliable results by evaluating the most recent and largest number of diseases that can be concurrently diagnosed from skin lesions. A randomly selected sample image for each class in the dataset is shown in Figure 1, along with the class label.

The dataset used in our study contains a total of 755 skin lesion images. These skin lesions, which were used in deep learning architectures, were obtained from 541 different patients. The dataset used to solve the multi-class classification problem contains images of 6 different diseases. Table 1 shows the number of images belonging to the diseases in the dataset and the number of patients from which these images were taken.

**Figure 2:**
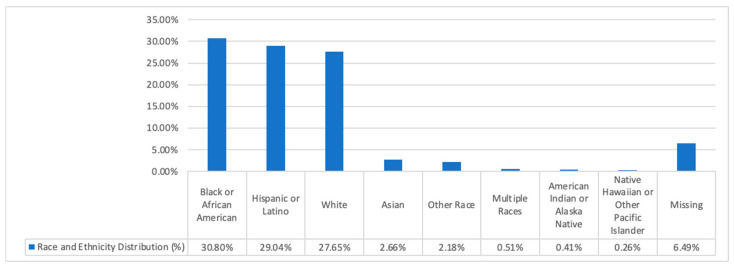
Distribution of patients in the MSLD v2.0 dataset according to race and ethnicity.

The dataset used in our study reflects different regions of the world in terms of demographic structure. The dataset also contains images of people from different ethnicities and races, such as Black, White, Latino, and Asian. Figure 2 shows the distribution of patients in the MSLD v2.0 dataset according to race and ethnicity. As can be seen in the figure, the dataset contains images of people from different parts of the world. This means that we are working with an inclusive dataset, which shows the strength of our study. The dataset was also demographically analyzed by dividing it into 6 different categories, from Type-1 (pale white) to Type-6 (very dark brown to black), on the Fitzpatrick skin tone scale. The dataset used in our study has 31.5%, 15.4%, 9.8%, 2.8%, 12.6%, and 28% distributions in Type-1, Type-2, Type-3, Type-4, Type-5, and Type-6 categories, respectively. The results obtained in the Fitzpatrick skin tone scale also confirm that the dataset can reflect different races and geographies. It was also stated by the creators of the dataset that the images in the dataset have different age and gender distributions, but since statistical information on these subjects was not clearly shared, they could not be examined in our study.

### 2.2. Vision Transformer (ViT)

Vision transformer (ViT) is a pioneering architecture that aims to utilize the superior capabilities of transformer-based architectures, which have shown outstanding success in capturing relationships between texts and feature extraction from texts in the field of image processing [15]. ViT architecture has been used by researchers to solve many problems in image processing, such as medical images, agriculture, and engineering. Compared to convolutional neural network (CNN) models, the ViT architecture has been able to achieve superior performance in many studies [16]. The most important advantage of the architecture is its flexible and modular structure. In this way, it is preferred by researchers in fields such as medicine. The prominent disadvantage of the architecture is its high memory usage and computational cost.

**Figure 3:**
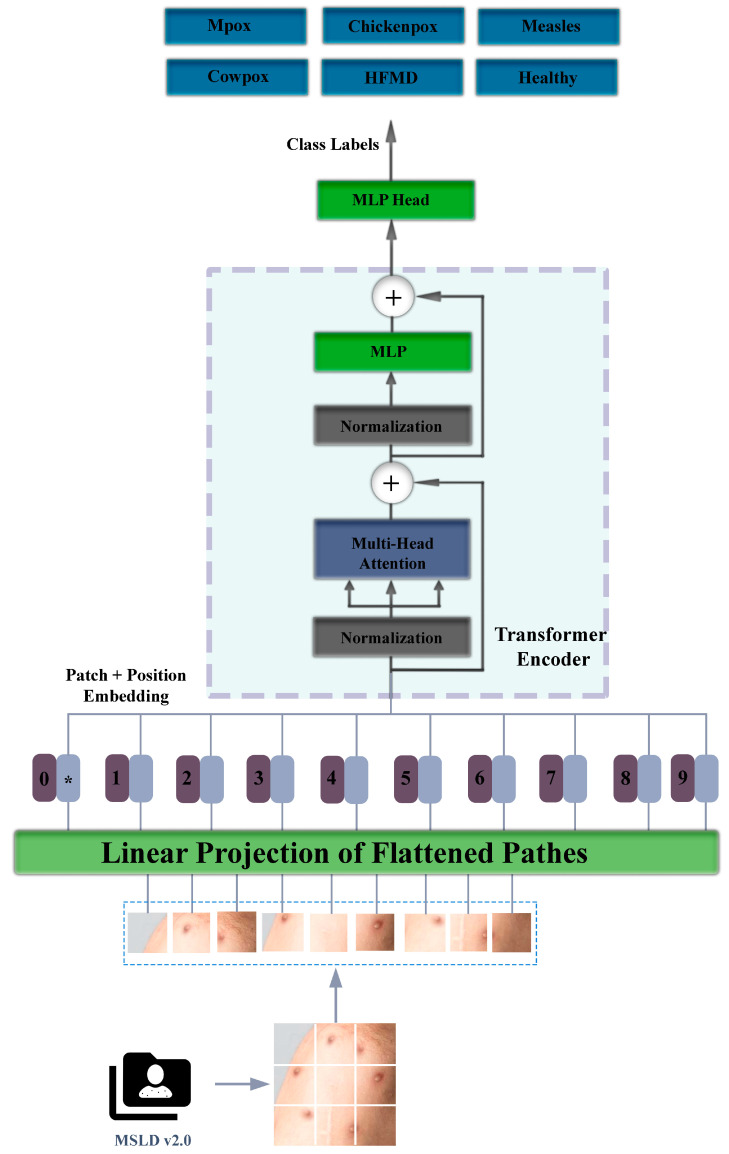
Vision transformer (ViT) model diagram.

ViT architecture starts with the subdivision of images into patches. Dividing the image into patches allows for more successful capture of distant relationships within the image. Pre-determined patches of 16 or 32 are used in the architecture. Position overlays are added to the image patches at the next stage, and they are passed through a smoothing layer [17]. They are processed in the normalization, multi-head attention, normalization, and MLP layers, respectively. In other words, the features of the patches are extracted using the attention mechanism, and MLP is applied to the resulting smoothed matrices. The part where these operations are performed is called the transformer encoder [18]. In the decoder part of the ViT architecture, class labels are obtained using the representations obtained in the encoder part. The block diagram of the vision transformer architecture used in our study is given in Figure 3.

### 2.3. Masked Autoencoders Are Scalable Vision Learners (MAE)

Masked Autoencoders Are Scalable Vision Learners is an important and revolutionary architecture that has emerged in recent years and can be used to solve problems such as the classification and segmentation of images [19]. The architecture, called MAE for short, offers an effective problem-solving capability within the framework of self-supervised learning. The architecture, which is developed based on vision transformer, can extract efficient features from medical images using the masking technique and increase classification success [20].

**Figure 4:**
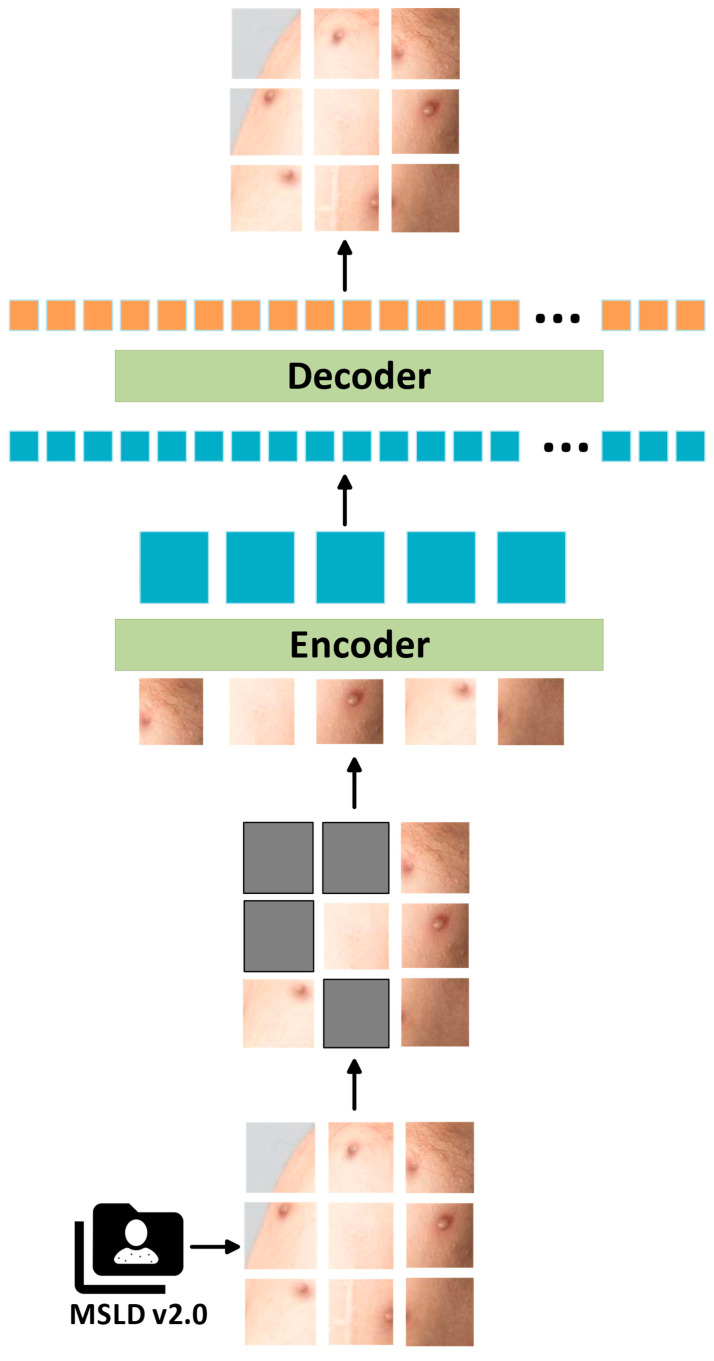
Masked Autoencoders Are Scalable Vision Learners (MAE) architecture block diagram.

MAE uses an encoder–decoder approach in its classification architectures. In the first stage of the architecture, images are divided into patches and made ready for processing in the architecture. Afterward, a large part of the image is masked. Using the masked images, the encoder–decoder architecture tries to learn the main representations of the image. In the encoder part, a small part of the image is presented as input, and these patches are encoded using ViT [21]. The masked image patches are not seen by the model at this stage. In the decoder stage, empty tokens are added instead of masked patches. The task of the decoder stage is to estimate these small image patches and recover the lost information. The most important part of the MAE architecture is the masking phase. The architecture randomly masks a large portion of the image patches [22]. The masked fraction is usually as large as 75%, meaning that a small fraction of the image, such as 25%, is used to recover the information of the full image. This increases the generalization performance by not allowing the model to take a wider view of the images. Another important advantage of the MAE model is scalability. The architecture can scale with more data and computational power with the benefit of ViT blocks. In addition, the fact that the architecture conducts the training process on masked paths reduces the memory requirement and provides efficient computational performance. A representative representation of our architecture applied to the MAE skin lesions dataset is given in Figure 4.

### 2.4. Emerging Properties in Self-Supervised Vision Transformers (DINO)

Self-supervised learning (SSL) is a deep learning approach in which the learning action is performed using the internal dynamics of the model without using class labels in the datasets [23]. In recent years, more efficient and highly successful models have been developed using SSL approaches in deep learning models. The Self-Distillation with No Labels (DINO) architecture is a hybrid and innovative approach that combines the SSL approach and ViT architecture. The DINO architecture has the capacity to achieve high classification performance even on limited data using a self-learning approach without a supervisor [24]. When working with limited data, such as the problems we focus on in our study, the DINO architecture can achieve significant success.

DINO is a transformer-based architecture that uses self-distillation. It includes student and tutor models; the student model is trained by emulating the output of the tutor model. The important difference from other architectures using distillation methods is that the tutor model is not updated using an external controller. The architecture updates its internal dynamics using the information obtained from the previous learner. Among the important advantages of the architecture are that it can achieve high classification performance thanks to its self-learning of generalizable representations, it has a flexible structure that can be used for different tasks, and it can be fine-tuned with transfer learning methods [25].

In the DINO architecture, which includes two separate networks, one with a student model and one with a teacher model, the models work on learning the same representation with different angles and slices [26]. While the teacher model is stable and static, the student model tries to resemble the teacher with updates. The overall goal of the architecture is to obtain generalizable embedded representations without using any classification header. The fact that it does not have to work with labeled data also reduces the need for a lot of data.

### 2.5. Hierarchical Vision Transformer Using Shifted Windows (Swin Transformer)

Swin transformer is a deep learning architecture developed by a Microsoft research team that focuses on developing a more efficient and scalable architecture based on the vision transformer architecture [27]. It is successfully used in many areas, such as feature extraction from images, classification, segmentation, and object detection. One of the most important features that distinguishes the architecture from other transformer-based architectures is the use of shifted window. Shifted window is the division of the image into fixed-size window blocks, then processing the elements in these windows in the transformer layer, and finally changing the position of the window by applying a shifting operation [28]. Another important innovation of the Swin transformer architecture is the hierarchical processing of images in which images are first divided into small parts, and then the windows are combined to extract large-scale features [29].

Another important feature of the Swin transformer architecture that distinguishes it from other transformer-based architectures is that it is a computationally efficient architecture. In traditional transformer-based architectures, the self-attention mechanism requires interaction between all array elements, and the computational cost increases significantly for large images. The implementation of a window-based attention mechanism significantly overcomes the computational cost problem in traditional transformer-based architectures [30]. The disadvantage of the Swin transformer architecture is the representativeness problem that may arise due to the fixed window size within the architecture. Large-scale objects may not fit in small windows within the architecture, which may cause a decrease in classification success.

**Figure 5:**
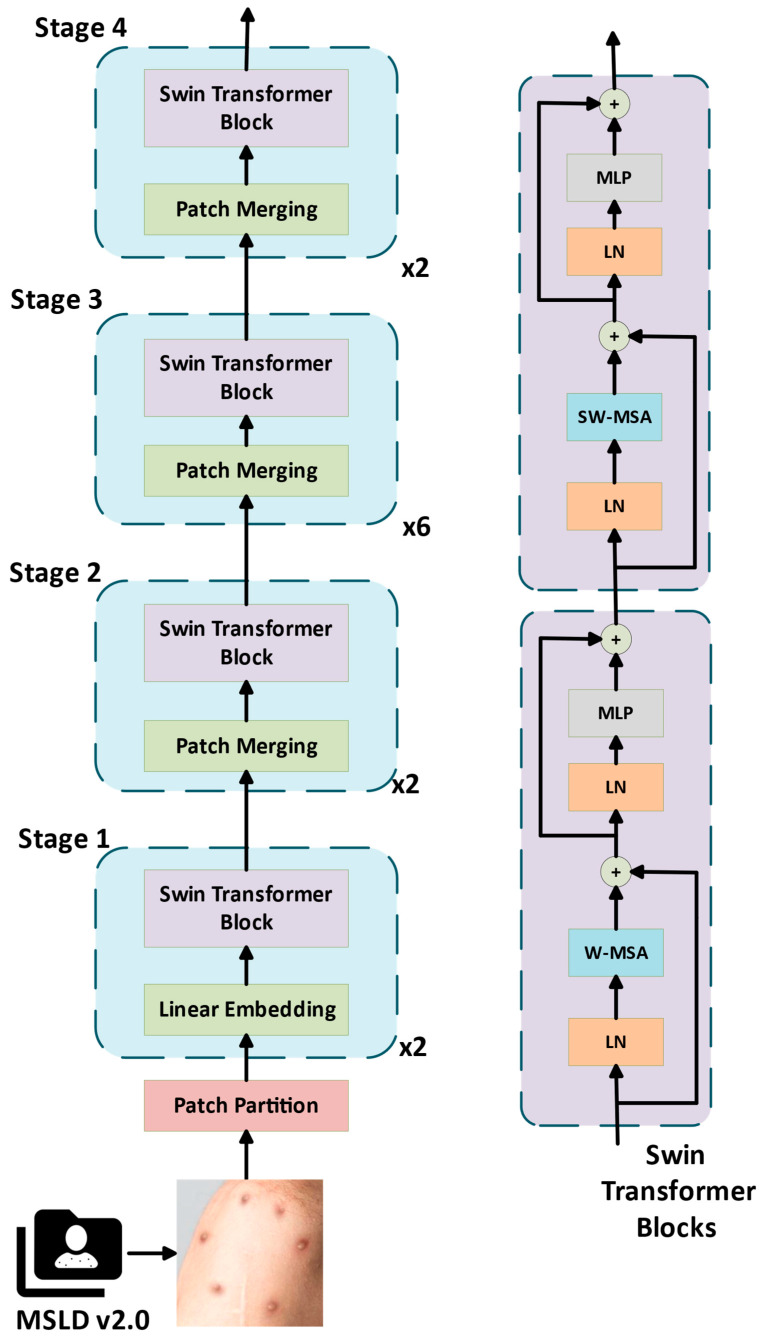
Hierarchical Vision Transformer Using Shifted Windows (Swin Transformer) architecture block diagram.

The Swin transformer architecture starts by dividing the input images into small windows and then applying self-attention within each window. The window is then shifted and information is transferred between the windows to learn the relationship between them. After the window-shifting process, hierarchical fusion is performed, and the obtained features are transmitted to the output layer [31]. In the output layer, the classification task is completed by using the feature maps in accordance with the number of classes. Figure 5 shows a representative block diagram of the Swin transformer architecture for the classification of skin lesion images.

### 2.6. Evaluation Metrics

Evaluation metrics are used to measure the classification success of deep learning architectures with measurable parameters. In our study, evaluation metrics were used to analyze how well the architecture performed in the testing phase for multi-class skin lesions, including Mpox disease, which we focused on in our study. The evaluation metrics are used to show how well the architecture performs in the research. Accuracy is the most commonly used evaluation metric and is calculated as the ratio of correct predictions to total predictions [8]. The accuracy metric provides a measurable parameter for the density of correct predictions of the architecture. Precision, recall, and F1 score evaluation metrics are other important metrics used to evaluate model performance. These are calculated using the confusion matrix. Precision, recall, and F1 score are also important evaluation metrics used in image classification in healthcare [32]. These evaluation metrics are measurable parameters calculated using the confusion matrix used to measure model performance. A confusion matrix is an important form of class-based representation of prediction performance using test data [33]. The confusion matrix provides a framework for the calculation of evaluation metrics.

## 3. Results

In order to observe the classification success of skin lesion images, including Mpox disease, with transformer-based deep learning architectures, a training and test environment was prepared using the Python programming language. All the architectures studied in the experimental environment were trained using a P100 GPU. The Kaggle platform was used as the development environment for faster and more reliable execution of deep learning codes. Kaggle is a developer platform by Google that allows researchers to run Python codes and also offers significant GPU support. P100 GPU was selected from the Kaggle platform at the training stage and the experimental environment was prepared. The batch size was 16 and the optimization algorithm was Adam. In all algorithms, the learning rate was chosen as 2 × 10⁻⁵. ImageNet pre-trained weights were used in the study. This feature is intended to improve model generalization performance when working with limited data. Training models from scratch requires much higher computational power, and the ImageNet weights used to overcome this disadvantage significantly reduce the training time of the model. In addition, the use of ImageNet weights is an important advantage in areas with limited data sources, such as medical image analysis. The subject of our study is also a new field of study with limited data. ImageNet weights were used in our study to improve performance, achieve faster training times, and improve generalization capability. The hyperparameters used in the studies are given in Table 2.

One of the most important representations of the success of our classification study with skin lesion images in the test results is the confusion matrix. The confusion matrix is the prediction of the model using test images that are not used in the training and validation processes, and the results obtained are placed in a dimensional matrix. The number of dimensions of the matrix is equal to the number of classes in order to represent all classes. The confusion matrix provides an environment for the calculation of other evaluation metrics. In addition, for each transformer-based architecture studied, class-based precision, recall, and F1 score evaluation metrics were also calculated. Table 3 shows the confusion matrix consisting of the class representations obtained for the vision transformer architecture, as well as the class-based classification results for the precision, recall, and F1 score evaluation metrics.

Classes 0, 1, 2, 3, 4, and 5 in the table represent Mpox, Chickenpox, Measles, Cowpox, HFMD, and a Healthy class, respectively. The labels P, R, and F1 stand for precision, sensitivity, and F1 score evaluation metrics, respectively. The researchers who created the data set published the images by dividing them into 5 different folds. Therefore, all experiments were conducted using a similar approach. By using a similar approach, the classification success of our study could be compared with other studies in the literature. For this reason, the dataset was divided into five folds, and each fold was analyzed separately and averaged. The data presented in Table 3, Table 4, Table 5 and Table 6 were obtained by taking the arithmetic mean and applying normalization to the results obtained from the 5-fold analysis. Table 4 shows the normalized confusion matrix and classification results obtained from the MAE architecture trained on skin lesion images.

When the disease-based results are analyzed, it is observed that the vision transformer architecture performs better than the MAE architecture with the addition of self-supervised learning and masking features. For Mpox disease with a 0 class label, the ViT architecture prediction was about 8% better than the MAE architecture prediction—while the true positive values for Mpox disease were 0.79% in the ViT architecture, this rate decreased to 0.71% in the MAE architecture. The precision evaluation metric labeled with P in the tables is used in the medical field, especially in cases where false positives should be low. Relying on high results for this metric is important to prevent false positives when diagnosing a serious disease. Diagnostic cost in the medical field is minimized by using this metric. When the values obtained in the test phases of MAE and ViT architectures are compared, it is seen that the ViT architecture is ahead, with a significant difference of 31% in the precision evaluation metric. In addition to the ViT and MAE architectures, training and testing processes were also carried out using the DINO architecture. In Table 5, the results obtained in the test phase of the DINO architecture are shared on a class basis and as a classification report.

When the results of the DINO deep learning architecture are analyzed, it is observed that it achieves better results than the MAE architecture. The DINO architecture achieved 28% higher Mpox disease diagnosis success with the precision evaluation metric compared to the MAE architecture. There is a difference of about 3% in the precision evaluation metric between the results obtained by the DINO architecture and the results obtained by the ViT architecture when classifying Mpox images. In this context, it can be said that DINO and ViT architectures achieve close results when classifying Mpox images. SwinTransformer architecture, which is a transformer-based architecture using a shifted window, was also used in our study. The class-based normalized confusion matrix and classification report for the test results obtained after training with the SwinTransformer architecture are given in Table 6.

When evaluated specifically for Mpox disease, it is seen that ViT and SwinTransformer architectures achieve similar results. When other skin lesions are evaluated together, it is observed that the SwinTransformer architecture achieves more inclusive results and achieves higher classification success than the ViT architecture. When Chickenpox disease, which is labeled as class 1 in the dataset, is examined, it is seen that the SwinTransformer architecture achieves 100% precision classification success, while the ViT architecture achieves 93% classification success.

**Figure 6:**
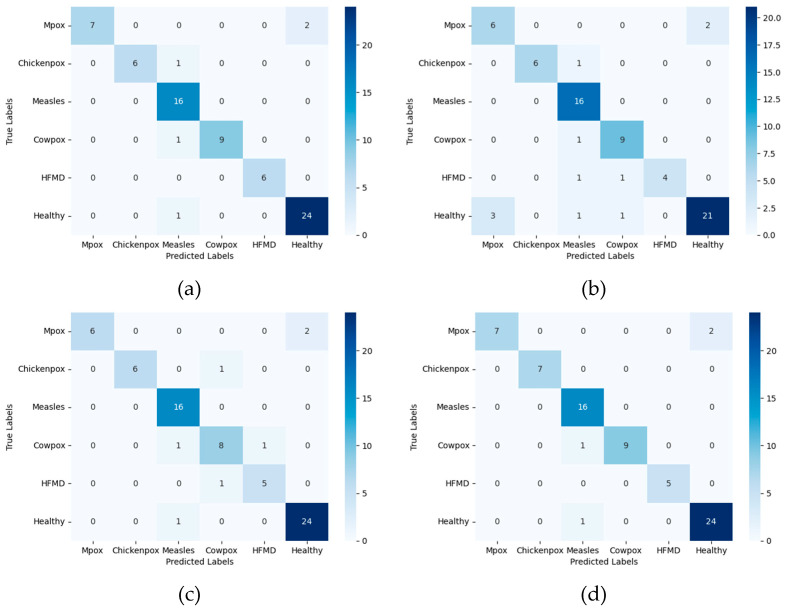
Confusion matrix test results for multi-class skin lesion images: (**a**) Vit, (**b**) MaeVit, (**c**) DinoVit, (**d**) SwinTransformer.

The confusion matrix is an important representation in machine learning and deep learning architectures that is used to measure their performance. Figure 6 shows the complexity matrices of four different deep learning architectures (ViT, MaeViT, DinoViT, and SwinTransformer) on multi-class skin lesion images. In order to more accurately compare the performances of deep learning architectures with existing studies in the literature, preserve the original structure of the dataset, and increase the confidence index, the datasets were divided into folds. The confusion matrix given in the figure was formed by taking the arithmetic mean of the folds studied. The confusion matrix visualizes the classification performance of the relevant model and provides a basis for calculating correct classifications and error rates. High values in the diagonal data in the complexity matrix mean that the model is correctly classified for the relevant classes. In all four models, diagonal elements are dominant and the classification performance of the architectures is high. In the SwinTransformer deep learning architecture, which has the highest disease diagnosis success, there are only four images that are outside the diagonal axis, that is, incorrectly predicted.

## 4. Discussion

This study was carried out to investigate the success of transformer-based deep learning architectures in classifying images of skin lesion diseases, including images of Mpox disease. In our study, ViT, MAE, DINO, and SwinTransformer architectures were used to train skin lesion images, and the results were analyzed in detail. In this section, the success and efficiency of our study will be analyzed by comparing the architectures used in the experiments with the results of other studies in the literature that used the same dataset.

The vision transformer (ViT) is a pioneering architecture in the field in which transformers, which have achieved significant success in text processing, are applied to image processing. However, over a short time, researchers have made many additions to the ViT architecture and, in some aspects, have created more powerful architectures. This study aims to analyze the effectiveness of four different transformer-based architectures based on their strengths and weaknesses. ViT, MAE, DINO, and SwinTransformer architectures have been developed in recent years and have been used in numerous papers worldwide.

Within the scope of the study, the four different transformer-based deep learning architectures were compared with other studies in the literature trained using similar parameters. Each of the transformer-based deep learning architectures selected in the study was chosen by the research team due to its different features. The ViT architecture is an important architecture preferred by many researchers who first used transformer architectures for image processing.

The MAE architecture is an architecture that has been extensively studied by researchers recently, where ViT architecture is improved with self-supervised learning and masking techniques. The DINO architecture is a hybrid, innovative, and groundbreaking architecture that combines self-distillation and self-supervised learning approaches with vision transformer architecture. The SwinTransformer architecture is an important architecture that has a reputation for achieving high success in many areas, powered by sliding windows and window-based self-attention mechanisms. Using the aforementioned features, the effect of the transformer-based architectures used in the study on the success of important innovations in the field on the classification of Mpox disease is also examined.

There are also important metrics that influence the choice of deep learning models, revealing which model is more efficient. These metrics evaluate important parameters, such as computational complexity, storage requirements, learning capacity, and speed, and guide researchers in model selection. FLOPs are a measure of the computational load of the model and represent the total floating point operations that the model undergoes while processing the input. Model size refers to the storage requirement of the model and represents how much disk space the model takes up. The model size parameter also determines the usability of the model on memory-constrained devices. The parameter count indicates the complexity and learning capacity of the model. Increasing the parameter count not only improves model performance but also requires more computational power and memory. Although models with small parameters achieve lower performance, in some cases, they may be preferable for memory-constrained or mobile devices. Inference time is defined as the time it takes to process an input and then generate a prediction. This metric can be critical for real-time applications. Table 7 shows the results of model parameters, model size (MB), FLOPs (GMac), and inference time (ms) model evaluation parameters obtained during the experiments on ViT, MaeViT, DinoVit, and SwinTransformer deep learning architectures.

In the literature, there are only two studies that focus on disease diagnosis from Mpox images and address the problem as a multi-class classification problem. Addressing the problem as a multi-class classification problem is a more difficult problem to solve. In order to diagnose other skin lesions along with Mpox and to propose an overarching framework that can support doctors in decision-making, the problem is considered a multi-class classification problem. Table 8 shows a comparison of the architectures in which we conducted training, validation, and testing processes within the scope of our study, as well as similar studies in the literature using the same evaluation metrics.

In the original version of the dataset, the test images were fold-sorted in five separate folders and published publicly. In order to accurately compare the results given in Table 7 with other studies in the literature, the test process was carried out without disturbing the assumed flod structure of the dataset, and weighted averages were taken and compared with other studies.

In the article describing the BinaryDNet53 architecture given in Table 8, it was not clear whether the pretrain network was used or not, so it could not be added to the table. In Ali et al. [10], the recall evaluation metric and results are not shared, and the confusion matrix shared in the study is normalized. For this reason, data on the recall evaluation metric could not be added in the first two rows of the table. When the results obtained with the accuracy evaluation metric are compared, the SwinTransformer architecture proposed in our study achieved the highest classification success (93.71%). The ViT architecture, on the other hand, achieved 0.6% lower accuracy evaluation metric success than the SwinTransformer architecture. DINO and MAE architectures ranked third and fourth in terms of classification success. In the literature, there are two studies published in 2024 that try to classify images of Mpox disease as a multi-class classification problem. The test results obtained in the experiments conducted within the scope of our study had much higher classification success compared to existing studies in the literature. While the BinaryDNet53 architecture, which has the highest success rate in the literature addressing the problem as a multi-class classification problem, achieved 85.78% accuracy, the SwinTransformer architecture proposed in our study achieved 93.71% accuracy. This shows that the proposed work achieves about 8% higher classification success than its competitors in the literature.

Table 8 compares the results obtained from the four deep learning models on which the experiments were performed with the studies in the literature using the Mpox Skin Lesion Dataset Version 2.0 (MSLD v2.0). The reason for choosing this dataset is that it is the dataset that approaches the problem from the widest perspective. The dataset we used in our study approaches skin lesions, including Mpox, as a six-class classification problem. In our study, there are six different disease classes: Mpox, Chickenpox, Measles, Cowpox, HFMD, and Healthy. A solution to the problem of autonomous diagnosis of these diseases using transformer-based deep learning architectures is sought. When the literature is examined, there are some studies that deal with Mpox disease as a two-class classification problem that only approaches Mpox disease as disease present and disease absent [34]. There are also some studies that deal with skin lesions, including Mpox disease, as a four-class classification problem [35]. Two and four-class classification studies evaluate the subject with a lower number of classes and work on older datasets. In this study, a newer set published in the second half of 2024 was used, and a comparison was made with studies using this dataset, which constitutes the limitations of our study in this context.

SwinTransformer has the highest success on the six-class classification problem, with an accuracy of 0.9371; however, this success is also directly related to the fact that the architecture has 86,749,374 parameters. The high number of parameters in the architecture is directly related to the higher learning capacity of the model and, thus, the higher success rate. On the other hand, the high number of parameters increases the computational cost. For this reason, researchers working on disease diagnosis from medical images should evaluate computational load and classification success together when choosing a transformer-based deep learning model. SwinTransformer is an innovative deep learning architecture that divides the attention mechanism into local sliding windows, but its computational cost is high compared to other popular transformer-based architectures. In healthcare applications, misdiagnosis or underdiagnosis of diseases can sometimes lead to disease progression and permanent harm to the patient. In some cases, incorrect or incomplete diagnoses can also lead to an undesirable situation, such as the loss of the patient’s life. For this reason, it is very important to have high diagnostic success in diagnostic applications in medical fields. For these reasons, experiments have shown that SwinTransformer, one of the popular transformer-based architectures, achieves the highest classification success with the highest computational cost in skin lesion images, including Mpox disease.

Mpox Skin Lesion Dataset Version 2.0 (MSLD v2.0), a new dataset published in the second semester of 2024, was used in this study. The fact that the images in the dataset are prepared in accordance with the multi-class classification problem and that it is the most up-to-date dataset containing Mpox disease is an important positive aspect that distinguishes the dataset from others. The training, validation, and testing processes of the study were carried out using the images in this dataset. In future studies, the researchers plan to collect their own data and expand their experiments by obtaining ethics committee permission. However, the images in the Mpox Skin Lesion Dataset Version 2.0 (MSLD v2.0) used in this version of the study constitute the limits of the study.

The dataset used in our study includes a total of 755 different images obtained from 541 patients. More than one image was obtained from some patients and used in the dataset. However, the process of obtaining multiple images from a patient was not computer-aided with image enhancement algorithms. As is known, skin lesions can spread to different organs and different surfaces of the body. Multiple images obtained from one patient were created by using images taken from different body parts of the patients. The same images were not used in the training, validation, and testing processes of the dataset, and the reliability of the study was considered. The use of the MSLD v2.0 dataset containing 755 different images from 541 different patients constitutes the limits of our study.

## 5. Conclusions

The main purpose of this study was to present a framework for autonomous disease diagnosis with transformer-based deep learning methods by treating skin lesion images, including Mpox disease, as a multi-class classification problem. The Mpox Skin Lesion Dataset Version 2.0, which was publicly released in 2024, was used for training, validation, and testing. ViT, MAE, DINO, and SwinTransformer architectures, which have been preferred by many researchers in recent years and have achieved high success in many studies, were trained for disease diagnosis from skin lesion images. The results obtained are analyzed in detail and shared in the publication. In the default version of the dataset, the test procedures were carried out by sticking to the 5-fold division of the test images, so that an accurate comparison with previous papers in the literature could be made. The SwinTransformer architecture we proposed in our study achieved about 8% higher accuracy evaluation metric classification success compared to its closest competitor in the literature. ViT, MAE, DINO, and SwinTransformer architectures achieved 93.10%, 84.60%, 90.40%, and 93.71% classification success, respectively.

The main contribution of the study to the literature is to investigate the success of transformer-based deep learning architectures in classifying Mpox disease as a multi-class classification problem. Rapid diagnosis of Mpox disease, which is an important health problem faced by the world after the COVID-19 pandemic, is important to prevent the spread of the disease. This study contains important results as it can detect Mpox disease among multi-class skin lesions with high success compared to other studies in the literature.

The limitations of our study are the use of multi-class skin lesion images of Mpox disease and the use of transformer-based architectures. The two-class classification problem was not included in the comparison tables by the research team because the coverage of the studies that addressed the problem as a two-class classification problem was low, and it was not a real-life problem. In addition, the studies examined in the literature are CNN-based studies. Transformer-based architectures were preferred in our study in order to eliminate the gap in the literature and to reveal the success of transformer-based architectures. In future studies, research will be conducted on new hybrid transformer-based architectures enhanced with distillation techniques to improve classification success.

## Figures and Tables

**Table 1:** Distribution of images in the MSLD v2.0 dataset based on skin lesions and number of patients.

Skin Lesion Class	Image Counts	Unique Patient Counts
Chickenpox	75	62
Cowpox	66	41
Healthy	114	105
HFMD	161	144
Measles	55	46
Mpox	284	143
Total	755	541

**Table 2:** Hyperparameters used in deep learning architecture.

Hyperparameter	Value
Batch Size	16
Learning Rate	2 × 10⁻⁵
Optimizer	Adam
Train Epochs	10
Pre-Trained	ImageNet

**Table 3:** Classification results of test images: predictions and classification report using vision transformer model.

		Predicted Class						Classification Report		
		0	1	2	3	4	5	P	R	F1
**Actual Class**	**0**	0.79	0	0	0	0	0.21	1.00	0.79	0.87
	**1**	0	0.86	0.14	0	0	0	0.92	0.83	0.86
	**2**	0	0.86	0.14	0	0	0	0.88	0.99	0.93
	**3**	0	0	0.06	0.94	0	0	0.95	0.93	0.94
	**4**	0	0	0	0	0.93	0.07	1.00	0.99	0.99
	**5**	0	0.01	0.02	0.01	0	0.96	0.93	0.96	0.94

**Table 4:** Classification results of test images: predictions and classification report using MAE model.

		Predicted Class						Classification Report		
		0	1	2	3	4	5	P	R	F1
**Actual Class**	**0**	0.71	0.05	0	0	0.02	0.21	0.69	0.70	0.69
	**1**	0	0.86	0.11	0	0	0.03	0.97	0.83	0.85
	**2**	0	0.86	0.11	0	0	0.03	0.85	0.98	0.91
	**3**	0	0	0.08	0.88	0	0.04	0.79	0.86	0.81
	**4**	0	0	0.10	0.17	0.67	0.07	0.94	0.68	0.77
	**5**	0.10	0	0.02	0.05	0	0.82	0.89	0.81	0.84

**Table 5:** Classification results of test images: predictions and classification report using the DINO model.

		Predicted Class						Classification Report		
		0	1	2	3	4	5	P	R	F1
**Actual Class**	**0**	0.74	0	0.05	0	0	0.21	0.97	0.72	0.82
	**1**	0	0.86	0.03	0.09	0	0.03	0.98	0.83	0.86
	**2**	0	0.86	0.03	0.09	0	0.03	0.89	0.99	0.94
	**3**	0	0	0.06	0.85	0.09	0	0.83	0.88	0.85
	**4**	0	0	0.07	0.10	0.80	0.03	0.95	0.85	0.90
	**5**	0.01	0.01	0.02	0.02	0	0.94	0.91	0.94	0.92

**Table 6:** Classification results of test images: predictions and classification report using the SwinTransformer model.

		Predicted Class						Classification Report		
		0	1	2	3	4	5	P	R	F1
**Actual Class**	**0**	0.79	0	0	0	0	0.21	1.00	0.79	0.87
	**1**	0	0.94	0.06	0	0	0	1.00	0.93	0.96
	**2**	0	0.94	0.06	0	0	0	0.89	0.99	0.93
	**3**	0	0	0.06	0.92	0.02	0	0.95	0.90	0.92
	**4**	0	0	0.07	0	0.90	0.03	0.96	0.90	0.93
	**5**	0	0	0.02	0.01	0	0.97	0.93	0.97	0.95

**Table 7:** Comparison of the performance metrics of transformer-based architectures in medical image classification.

Model	Model Parameters	Model Size (MB)	FLOPs (GMac)	Inference Time (ms)
ViT	85,806,346	327.40	16.87	6.84
MaeViT	85,803,270	327.39	16.87	6.83
DinoVit	21,667,974	82.73	4.25	6.93
SwinTransformer	86,749,374	331.54	15.47	28.73

**Table 8:** Comparative performance evaluation of deep learning architectures for classification of multi-class skin lesion images, including Mpox disease.

Model	Pre-Train	Accuracy	Precision	Recall	F1-Score
Ali et al. [10]	ImageNet	0.8170	0.8300	-	0.8000
Ali et al. [10]	Ham1000	0.8226	0.8500	-	0.8300
BinaryDNet53 [11]	-	0.8578	0.8692	0.8246	0.8420
ViT	ImageNet	0.9310	0.9360	0.9310	0.9275
MAE	ImageNet	0.8460	0.8626	0.8460	0.8429
Dino	ImageNet	0.9040	0.9152	0.9040	0.8989
SwinTransformer	ImageNet	0.9371	0.9428	0.9371	0.9358

## Data Availability

The data supporting the findings of this study are based on a publicly available dataset in the reference: Mpox Skin Lesion Dataset Version 2.0 (MSLD v2.0) at https://www.kaggle.com/datasets/joydippaul/mpox-skin-lesion-dataset-version-20-msld-v20/data (accessed on 20 November 2024).

## References

[1] Van Nispen C., Reffett T., Long B., Gottlieb M., Frawley T.C. (2023). Diagnosis and Management of Monkeypox: A Review for the Emergency Clinician. Ann. Emerg. Med..

[2] Khani E., Afsharirad B., Entezari-Maleki T. (2023). Monkeypox Treatment: Current Evidence and Future Perspectives. J. Med. Virol..

[3] Fowotade A., Fasuyi T.O., Bakare R.A. (2018). Re-Emergence of Monkeypox in Nigeria: A Cause for Concern and Public Enlightenment. Afr. J. Clin. Exp. Microbiol..

[4] Akkuzu N., Ucan M., Kaya M. Classification of Multi-Label Electrocardiograms Utilizing the EfficientNet CNN Model. Proceedings of the 2023 4th International Conference on Data Analytics for Business and Industry (ICDABI).

[5] Minaee S., Kafieh R., Sonka M., Yazdani S., Jamalipour Soufi G. (2020). Deep-COVID: Predicting COVID-19 from Chest X-Ray Images Using Deep Transfer Learning. Med. Image Anal..

[6] Tee M.L., Tee C.A., Anlacan J.P., Aligam K.J.G., Reyes P.W.C., Kuruchittham V., Ho R.C. (2020). Psychological Impact of COVID-19 Pandemic in the Philippines. J. Affect. Disord..

[7] Ucan S., Ucan M., Kaya M. Deep Learning Based Approach with EfficientNet and SE Block Attention Mechanism for Multiclass Alzheimer’s Disease Detection. Proceedings of the 2023 4th International Conference on Data Analytics for Business and Industry (ICDABI).

[8] Charoenchue P., Khorana J., Chitapanarux T., Inmutto N., Na Chiangmai W., Amantakul A., Pojchamarnwiputh S., Tantraworasin A. (2025). Two-Dimensional Shear-Wave Elastography: Accuracy in Liver Fibrosis Staging Using Magnetic Resonance Elastography as the Reference Standard. Diagnostics.

[9] Song H., Sun D., Chun S., Jampani V., Han D., Heo B., Kim W., Yang M.-H. (2022). An Extendable, Efficient and Effective Transformer-Based Object Detector. arXiv.

[10] Ali S.N., Ahmed M.T., Jahan T., Paul J., Sakeef Sani S.M., Noor N., Asma A.N., Hasan T. (2024). A Web-Based Mpox Skin Lesion Detection System Using State-of-the-Art Deep Learning Models Considering Racial Diversity. Biomed. Signal Process Control.

[11] Biswas D., Tešić J. (2024). Binarydnet53: A Lightweight Binarized CNN for Monkeypox Virus Image Classification. Signal Image Video Process..

[12] Bala D., Hossain M.S., Hossain M.A., Abdullah M.I., Rahman M.M., Manavalan B., Gu N., Islam M.S., Huang Z. (2023). MonkeyNet: A Robust Deep Convolutional Neural Network for Monkeypox Disease Detection and Classification. Neural Netw..

[13] Akram A., Jamjoom A.A., Innab N., Almujally N.A., Umer M., Alsubai S., Fimiani G. (2024). SkinMarkNet: An Automated Approach for Prediction of MonkeyPox Using Image Data Augmentation with Deep Ensemble Learning Models. Multimed. Tools Appl..

[14] Mpox Skin Lesion Dataset Version 2.0 (MSLD v2.0). https://www.kaggle.com/datasets/joydippaul/mpox-skin-lesion-dataset-version-20-msld-v20/data.

[15] Dosovitskiy A., Beyer L., Kolesnikov A., Weissenborn D., Zhai X., Unterthiner T., Dehghani M., Minderer M., Heigold G., Gelly S. (2020). An Image Is Worth 16x16 Words: Transformers for Image Recognition at Scale. arXiv.

[16] Maurício J., Domingues I., Bernardino J. (2023). Comparing Vision Transformers and Convolutional Neural Networks for Image Classification: A Literature Review. Appl. Sci..

[17] Han K., Wang Y., Chen H., Chen X., Guo J., Liu Z., Tang Y., Xiao A., Xu C., Xu Y. (2023). A Survey on Vision Transformer. IEEE Trans. Pattern Anal. Mach. Intell..

[18] Manzari O.N., Ahmadabadi H., Kashiani H., Shokouhi S.B., Ayatollahi A. (2023). MedViT: A Robust Vision Transformer for Generalized Medical Image Classification. Comput. Biol. Med..

[19] He K., Chen X., Xie S., Li Y., Dollár P., Girshick R. Masked Autoencoders Are Scalable Vision Learners. Proceedings of the 2022 IEEE/CVF Conference on Computer Vision and Pattern Recognition.

[20] Xu Z., Dai Y., Liu F., Chen W., Liu Y., Shi L., Liu S., Zhou Y. (2023). Swin MAE: Masked Autoencoders for Small Datasets. Comput. Biol. Med..

[21] Zhang H., Patkar S., Lis R., Merino M.J., Pinto P.A., Choyke P.L., Turkbey B., Harmon S. (2024). Masked Image Modeling Meets Self-Distillation: A Transformer-Based Prostate Gland Segmentation Framework for Pathology Slides. Cancers.

[22] Lu Q., Wang B., He Q., Zhang Q., Guo L., Li J., Li J., Ma Q. (2024). A Blood Cell Classification Method Based on MAE and Active Learning. Biomed. Signal Process Control.

[23] Caron M., Touvron H., Misra I., Jégou H., Mairal J., Bojanowski P., Joulin A. Emerging Properties in Self-Supervised Vision Transformers. Proceedings of the 2021 IEEE/CVF International Conference on Computer Vision.

[24] Kim J.-Y., Tangriberganov G., Jung W., Kim D.S., Koo H.S., Lee S., Kim S.M. (2023). An Effective Representation Learning Approach: The Integrated Self-Supervised Pre-Training Models of StyleGAN2-ADA and DINO for Colon Polyp Images. IEEE Access.

[25] Park S., Lee I.J., Kim J.W., Chul Ye J. (2024). MS-DINO: Masked Self-Supervised Distributed Learning Using Vision Transformer. IEEE J. Biomed. Health Inform..

[26] Wessels F., Schmitt M., Krieghoff-Henning E., Nientiedt M., Waldbillig F., Neuberger M., Kriegmair M.C., Kowalewski K.-F., Worst T.S., Steeg M. (2023). A Self-Supervised Vision Transformer to Predict Survival from Histopathology in Renal Cell Carcinoma. World J. Urol..

[27] Liu Z., Lin Y., Cao Y., Hu H., Wei Y., Zhang Z., Lin S., Guo B. Swin Transformer: Hierarchical Vision Transformer Using Shifted Windows. Proceedings of the 2021 IEEE/CVF International Conference on Computer Vision.

[28] Ferdous G.J., Sathi K.A., Hossain M.A., Dewan M.A.A. (2024). SPT-Swin: A Shifted Patch Tokenization Swin Transformer for Image Classification. IEEE Access.

[29] Huang J., Fang Y., Wu Y., Wu H., Gao Z., Li Y., Del Ser J., Xia J., Yang G. (2022). Swin Transformer for Fast MRI. Neurocomputing.

[30] Sankari V.M.R., Umapathy U., Alasmari S., Aslam S.M. (2023). Automated Detection of Retinopathy of Prematurity Using Quantum Machine Learning and Deep Learning Techniques. IEEE Access.

[31] Üzen H., Firat H., Atila O., Şengür A. (2024). Swin Transformer-Based Fork Architecture for Automated Breast Tumor Classification. Expert. Syst. Appl..

[32] Disci R., Gurcan F., Soylu A. (2025). Advanced Brain Tumor Classification in MR Images Using Transfer Learning and Pre-Trained Deep CNN Models. Cancers.

[33] Markoulidakis I., Rallis I., Georgoulas I., Kopsiaftis G., Doulamis A., Doulamis N. (2021). Multiclass Confusion Matrix Reduction Method and Its Application on Net Promoter Score Classification Problem. Technologies.

[34] Jaradat A.S., Al Mamlook R.E., Almakayeel N., Alharbe N., Almuflih A.S., Nasayreh A., Gharaibeh H., Gharaibeh M., Gharaibeh A., Bzizi H. (2023). Automated Monkeypox Skin Lesion Detection Using Deep Learning and Transfer Learning Techniques. Int. J. Environ. Res. Public Health.

[35] Al-Gaashani M.S.A.M., Xu W., Obsie E.Y. (2025). MobileNetV2-Based Deep Learning Architecture with Progressive Transfer Learning for Accurate Monkeypox Detection. Appl. Soft Comput..

